# Tick infestation of the eyelid

**DOI:** 10.1590/0037-8682-0599-2019

**Published:** 2020-06-12

**Authors:** Raghunandanan Rama Varma, Parvathi Varma, Anil Kumar

**Affiliations:** 1 Consultant Ophthalmologist, Krishna Hospital, Kochi, Kerala, India, Pin:682016.; 2 Research Scientist, Molecular Biology, Amrita Institute of Medical Sciences, Amrita Vishwa Vidyapeetham, Ponekara, Kochi, Kerala India.; 3 Department of Microbiology, Amrita Institute of Medical Sciences, Amrita Vishwa Vidyapeetham, Ponekara, Kochi, Kerala India.

A 57-year-old man, who was otherwise healthy, presented with complaints of a rapidly growing tumor on the lower eyelid of his right eye since the past two weeks. Using the slit lamp examination, a light brown smooth, glistening tick was found on the lower eyelid of his right eye (Figure 1). The tick was removed intact without breaking its mouth-parts (Figure 2). The patient had a pet dog, which upon examination was found to be infested with ticks of the same species. The Patient underwent regular follow-up; however, no signs and symptoms of any tick-borne diseases was observed.

**FIGURE 1:**
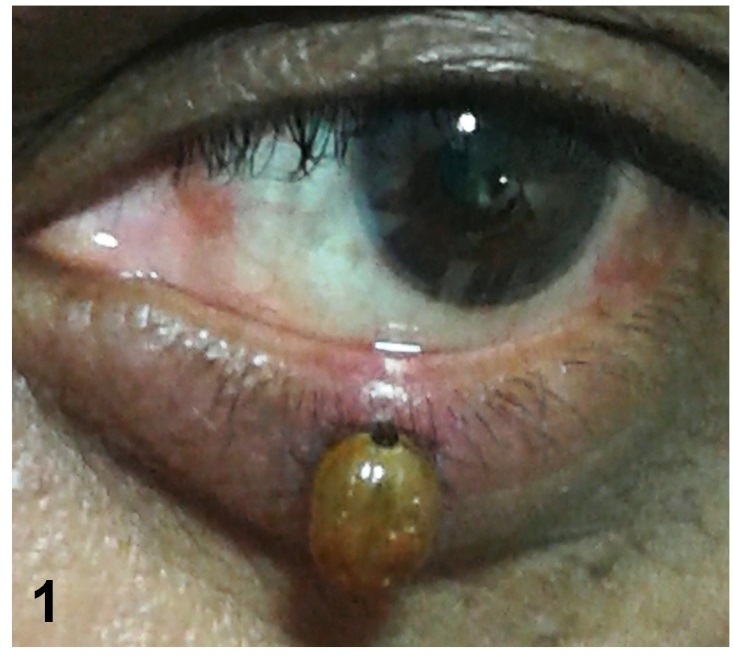
Brown dog tick attached to the lower eyelid.

The tick was identified as an engorged adult female (as males do not enlarge upon feeding) of the reddish brown dog tick (*Rhipicephalus sanguineus*) (Figure 2), measuring about 12 mm in length, with short and stout mouth-parts. The tick was distinguished from the American dog tick by its brown color and the absence of light wavy lines or reticulations on its back. Furthermore, the tick was distinguished from the lone star ticks by the absence of a central white spot on its back. Tick bites are known to transmit a variety of bacterial, viral, and parasitic diseases to humans. The brown dog tick is particularly associated with transmission of Rocky Mountain spotted fever, a life threatening, tick-borne disease caused by *Rickettsia rickettsia* ^1^. Another rare manifestation is tick paralysis, which may be caused by a toxin present in the tick saliva^2^.

**FIGURE 2:**
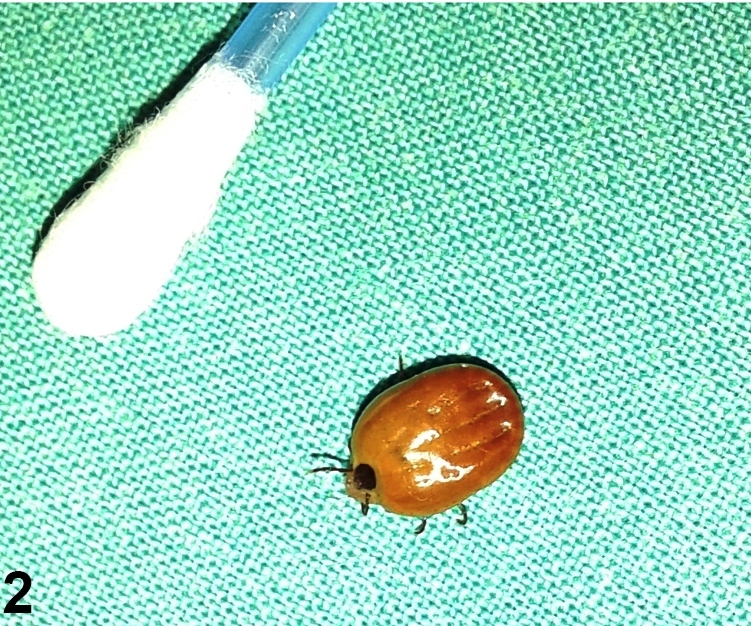
Brown dog tick after being removed from the eyelid.

Tick infestation of eyelids might have occurred due to a close contact with pets like dogs. Patient should be followed up to rule out any systemic illness transmitted by ticks.

## References

[1] Demma LJ, Traeger MS, Nicholson WL, Paddock CD, Blau DM, Eremeeva ME (2005). Rocky Mountain spotted fever from an unexpected tick vector in Arizona. N Engl J Med.

[2] Engin A, Elaldi N, Bolayir E, Dokmetas I, Bakir M (2006). Tick paralysis with atypical presentation: isolated, reversible involvement of the upper trunk of brachial plexus. Emerg Med J.

